# Supplementary material for: Multiomics Provide Insights into the Key Molecules and Pathways Involved in the Physiological Adaptation of Atlantic Salmon (Salmo salar) to Chemotherapeutic-Induced Oxidative Stress
Source: Antioxidants (Basel). 2021 Nov 30;10(12):1931. doi: 10.3390/antiox10121931 (PMC8750430; doi:10.3390/antiox10121931)
Supplement: Supplementary file 1 [file antioxidants-10-01931-s001.zip › Figure S1 Mucous cells.pdf]

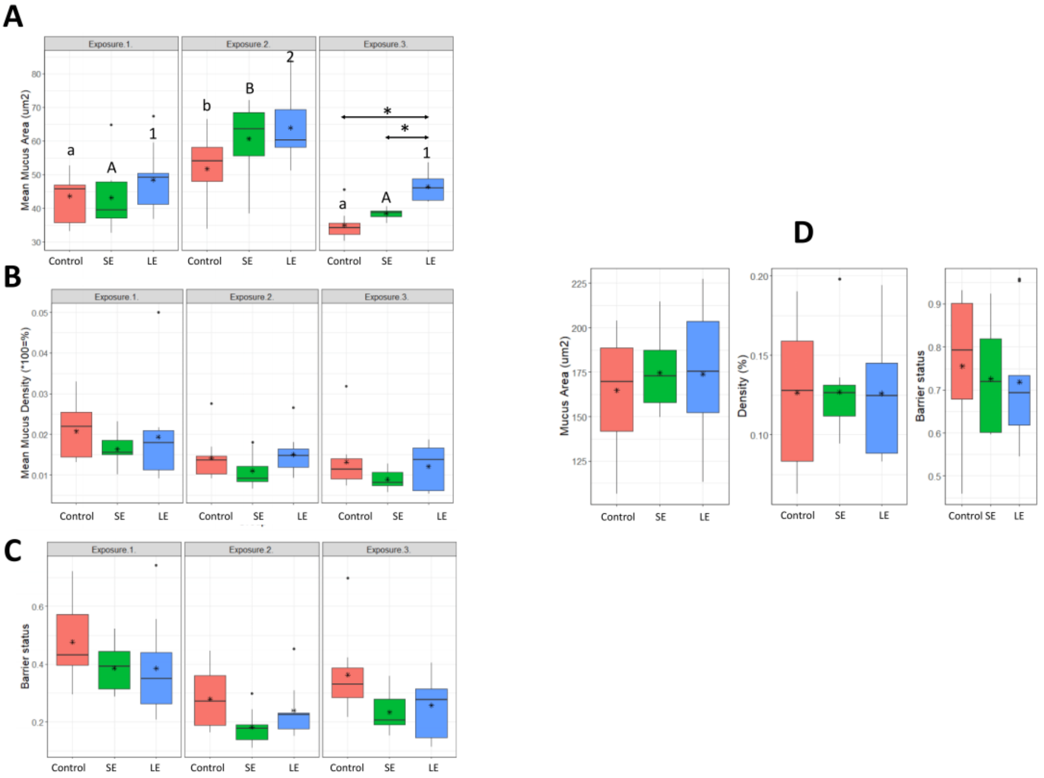

Supplementary Figure 1. Key mucosal morphometries of the gills and skin. (A–C) Gill samples were taken 24 h after each exposure, while (D) skin samples were only analysed after the 3rd exposure. Mean mucous area, density, and barrier status were measured using Quantidoc's mucosal mapping method. N = 9 fish per treatment group. Please refer to Fig. 1 for the statistical notations. SE, short exposure (15 min); LE, long exposure (30 min).
